# Ecological risk assessment of heavy metals in tea plantation soil around Tai Lake region in Suzhou, China

**DOI:** 10.1007/s44154-024-00149-x

**Published:** 2024-02-16

**Authors:** Xiaohan Xu, Jiahui Yang, Yang Zhang, Xueyan Sui, Zelong Gong, Shujing Liu, Xuan Chen, Xinghui Li, Yuhua Wang

**Affiliations:** 1 College of Horticulture, Nanjing Agricultural University, Nanjing, 210095 China; 2 Jiangsu Land Consolidation and Rehabilitation Center, Nanjing, 210017 China; 3 Jiangsu Donghai and Yixing Land Consolidation and Ecological Protection Field Scientific Observation and Research Station, Ministry of Natural Resources, Yixing, 214200 China

**Keywords:** Tea plantations, Soil, Heavy metal, Ecological risk assessment

## Abstract

Tea plant [*Camellia sinensis* (L.) O. Kuntze] is one of the important foliar cash crops in China, and its root system absorbs heavy metal (HM) elements enriched in the soil and transports them to the over ground part. In order to ensure the safety of the soil ecological environment and tea raw materials in the tea production area, the HM contents of soil and tea plant leaves in Suzhou tea plantations were detected, the relationship between HMs and soil physicochemical properties was analyzed, and the ecological risk of HMs in tea plantation soils was evaluated by using relevant detection techniques and evaluation models. The results showed that the average pH of tea plantation soils around Tai Lake in Suzhou was within the range suitable for the growth of tea plants. The pH, soil organic matter, total nitrogen, available phosphorus and available potassium of tea plantation soil satisfying the requirements of high quality, high efficiency and high yield (‘3H’) tea plantation accounted for 47.06%, 26.47%, 8.82%, 79.41% and 67.65%, respectively. Site 2 fully met the requirements of ‘3H’ tea plantation. In addition, the contents of cadmium (Cd) and mercury (Hg) were extremely variable, and the average contents exceeded the background value of soil in Jiangsu Province, but the HM contents of tea leaves all met the pollution-free standard, and the HM contents of tea leaves around Tai Lake in Suzhou were generally at a safe level. The composite ecological risk index ranged from 0.05 to 0.60, and 32 of the 34 sample sites (except site 21 and site 23) are the most suitable agricultural land for tea plantations.

**Supplementary Information:**

The online version contains supplementary material available at 10.1007/s44154-024-00149-x.

## Introduction

Any metal having a density of more than 4.5 kg dm^−3^ is commonly referred to as a HM in the industrial sector, and from the perspective of environmental protection, the most biologically harmful elements such as Cu, Pb, Cd, Zn, Hg, As, etc. are categorized as HM elements (Xu et al. 2019). HM accumulation in soil is a significant problem due to its long-term persistence, cunningness, laggardly character, and irreversibility (Xu et al. 2013; Massas et al. 2013). The quality of agricultural soils is gradually deteriorating due to the use of chemical fertilizers and pesticides in agricultural production activities, as well as the arbitrary discharge of sewage and manure from livestock and poultry (Guan and Sun 2014). As a result, soil HM overloading is becoming a significant environmental problem in China (Xiao et al. 2017). According to the ‘Bulletin on the Investigation of Soil Pollution in China’, the rate of soil HM exceedance was 16.1% (MEE and MNR 2014). In recent years, the tea plantations near the “three wastes” have continued to raise the ecological danger of HMs in the soil due to the influence of industrialization, in addition to air deposition, pesticides, fertilizers, and other reasons.

Tea plant [*Camellia sinensis* (L.) O. Kuntze] is an important cash crop in the subtropical region of China. According to the statistics of the “2021 China Tea Production and Marketing Situation Report” released by the China Tea Circulation Association, the total area of tea gardens in 18 major tea-producing regions in 2021 is 3,264,406 ha (about 60% of the global total tea garden area), and the total national output is 3.18 million tons (about 45% of the global total tea production). The annual production of green tea is 1.8494 million tons, accounting for 60.38% of the total production (Association 2022). In recent years, there have been more and more reports on the excess of HMs in tea (Ju et al. 2024; Gogoi et al. 2023; SeyyediBidgoli et al. 2020). According to popular belief, the soil in tea plantations serves as the plant’s physical foundation. Its elemental composition is also the primary source of HMs in tea, and it has a significant impact on the development, metabolism, production, and quality of tea plants (Othman et al. 2012; Lv et al. 2013; Xu et al. 2013; Guan and Sun 2014). The distribution of soil HMs is largely influenced by geological differences (Zhang et al. 2020b), and there is a high degree of correlation between soil physicochemical properties and HM elements (Yang et al. 2018), so that there are some differences in the HM contents of tea plantation soils in different regions.

HM enrichment in soil not only causes ecological damage and deterioration of environmental quality, but also affects human health through the food chain (Xu et al. 2019). Gradual enrichment of HMs or decomposition products in the environment and in the human body through the “soil-plant-body” system (Guan and Sun 2014). For microorganisms, bacterial communities such as *Acidobacteria* and *Bacillus* were highly resistant and more abundant in tea plantations with acidic soils and higher levels of HM exceedances compared to other bacterial communities (Lei et al. 2018). Additionally, the tea plant’s development condition will be significantly impacted by an overabundance of HM components. According to research findings, Cd stress makes tea plant leaves brown, green, or yellow while preventing the growth of buds, roots, and new shoots (Podwika et al. 2017). The effects of Hg stress on the tea plant’s cells and metabolism cause the plant to eventually wither or possibly die (Suszcynsky and Shann 1995). Drinking large amounts of HMs in tea over the long term can be harmful to one’s health.

Environmental ecological risk assessment is, in the words of the U.S. EPA, “the process of evaluating the likelihood of adverse ecological impacts that may occur or are occurring as a result of exposure to one or more stressors (e.g., chemicals, exotic species, physical changes, fire, etc.)” (Smith et al. 2005). The United States (Usepa. 1996), Australia (Council 2013) and other countries have adopted a hierarchical approach to build an ecological risk assessment framework for soil HMs, while the assessment framework of Canada (Ohio 2003) does not include a hierarchical assessment process, but in order to reduce the uncertainty of the assessment, it adds a screening assessment step prior to the ecological risk assessment process, and the risk manager or decision maker. The risk manager or decision maker determines the number of iterations of a step in the basic framework based on the actual needs of soil ecosystem management. The “weight-of-evidence” (WoE) method is widely used in soil ecological risk assessment, which is characterized by its systematic, holistic and scientific nature. Dagnino et al. (2008) proposed a chemical parameter based on HM concentration, an ecotoxicological parameter at the level of individual organisms, a biophysiological parameter at the level of sublethal HMs, and an ecological parameter related to the structure and function of the biota, which were compared with the corresponding reference values, and finally a comprehensive ecological risk index was obtained by using the WoE method according to the respective weights, which led to a comprehensive ecological risk assessment.

Suzhou City, one of the key regions for producing China’s renowned green tea, has a long history of producing tea. Jiangsu Province’s Suzhou Tai Lake, which makes up 14.3% of the entire province’s land area and has promising growth potential, is located along a large expanse of rugged mountainous landscapes. The hilly mountainous areas have been included in the “Ninth Five-Year Plan” and the “Notice on Accelerating Comprehensive Agricultural Development in the Hilly Mountainous Areas of the Province” since 1996. Since then, the provincial government has gradually strengthened resource development and utilization in the hilly mountainous areas, which has led to a rapid rise in the level of economic and social development. This study adopts the WoE method to study the HM risk of tea plantation soils around Tai Lake area in Suzhou by investigating, detecting and assessing the current status of HM content, analyzing the current problems, and then providing a theoretical basis for the reasonable and effective treatment and remediation of the HM risk of tea plantation soils in Suzhou City and Jiangsu Province. Effective governance and repair to provide a theoretical basis, and then promote the sustainable development of green ecological agricultural construction.

## Material and method

### Study area and sample collection

**Fig. 1:**
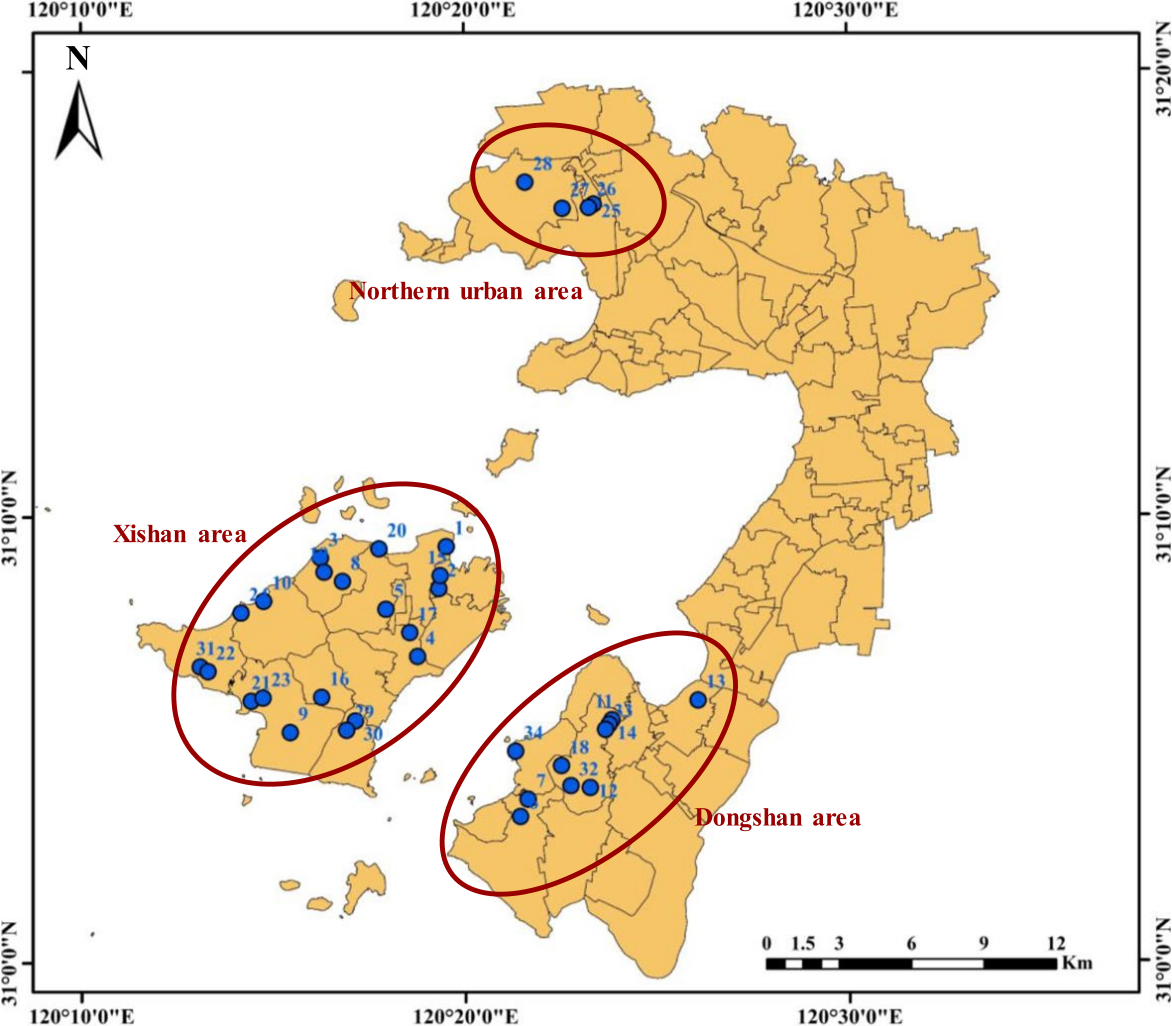
Location of the study area and sampling sites

Using ArcGIS 10.7 software, 34 sample sites were deployed in the tea plantations around Tai Lake area of Suzhou in accordance with the current land use situation in Wuzhong District, Suzhou City, as well as field research (Fig. 1). Each site was deployed with 3–5 sampling points depending on the size of the area, and 34 sites in total were deployed. After the surface litters and 0–20 cm of topsoil were removed, the soil samples were selected from 20 to 40 cm layer using a 3.5 cm-diameter soil auger in late September 2022. The soil samples, which were kept on ice in the field, were taken to laboratory then passed through 2 mm sieve after removing the fine roots and stones manually. The photos were taken to record the sampling depth of the sampling sites and the latitude and longitude of each sampling site were recorded by GPS after sampling. In the second half of March 2023, the young leaves (one bud and three leaves) and mature leaves of tea plant leaves were picked at the above 34 sites. The leaf samples, quick freezing with liquid nitrogen in the field, were brought back to the laboratory the same day and killed-green in a microwave oven for 15 min, then baked in an oven at 70 °C until constant weight, the tea samples were ground to powder and sieved through a 0.45 mm sieve, and the sieved tea powder was placed in a polyethylene self-sealing bag to be stored in a dry place and used for subsequent experiments.

### Determination of soil physical and chemical properties

Soil water content was determined by drying method with reference to Liu et al. (2018). Briefly, 50.00 g of fresh soil samples through 2 mm sieve were weighed in an aluminum box of known weight (accurate to 0.01 g), placed in an oven that had been preheated to 105 °C for 24 h, cooled to room temperature in a desiccator, the dry weight was determined, and the soil water content was calculated. Field water content (FWC) was determined by referring to the PRC Forestry Industry Standard (China 1999). Soil pH and organic matter (SOM) content were determined with reference to He et al. (2016).

The contents of NH_4_^+^-N and NO_3_^−^-N were determined by automatic continuous flow analyzer (seal AA3, Germany) (Zhang et al. 2020a). Briefly, 2.00 g of fresh soil samples with 2 mm sieve were weighed into 50 mL centrifuge tubes, 20 mL of 2 mol L^−1^ KCl was added to a shaker at room temperature for 1 h. The samples were filtered through qualitative filter paper and the filtrate was used for determination. Total carbon (TC) and total nitrogen (TN) contents were determined using an elemental analyzer (Jena multi EA 5000, Germany). Soil effective phosphorus available phosphorus (AP) content was determined using molybdenum antimony colorimetric method with reference to MOA (2014). Soil available potassium (AK) content was determined using an inductively coupled plasma mass spectrometer ICP-MS (Thermo Fisher Scientific, USA). Briefly, 2.5 g of air-dried soil passing a 1 mm sieve was weighed into a 50 mL centrifuge tube, 25 mL of 0.1 mol L^−1^ neutral ammonium acetate solution was added, and the solution was shaken for 30 min at room temperature, filtered through qualitative filter paper, and the filtrate was used for the determination.

According to the method of Li et al. (2019), weigh 0.1 g soil sample passing through 0.15 mm sieve, put it in a PTFE digestion tank, add 4.5 mL HCl and 1.5 mL HNO_3_, and put it in a microwave digestion instrument to digest the sample. After digestion, the solution was diluted to 50 mL with ultrapure water, and 10 mL of the diluted solution was filtered with a membrane with a diameter of 0.22 μm. The contents of TP and TK in the filtrate were determined by inductively coupled plasma mass spectrometer ICP-MS (Thermo Fisher Scientific, USA) (Jing et al. 2019).

### Determination of heavy metal content

According to the method of Li et al. (2019), weigh 0.1 g of soil sample with a sieve of 0.15 mm, put it in a polytetrafluoroethylene digestion tank, add 4 mL HNO_3_ and 1 mL HClO_4_, and put the mixed solution in a microwave digestion instrument to digest the sample. After digestion, the solution was diluted to 50 mL with deionized water, and 10 mL of the diluted solution was filtered with a 0.45 μm filter membrane. The heavy metal content in soil was determined by inductively coupled plasma mass spectrometer ICP-MS (Thermo Fisher Scientific, USA) (Jing et al. 2019).

According to the method of Xu et al. (2019), 0.2 g of tea plant leaf samples were weighed in a polytetrafluoroethylene digestion jar. The samples were digested with a mixture of 8 mL of HNO_3_ and 2 mL of HClO_4_, and the samples were digested at 150 °C until the solution was colorless and transparent. The digested solution was then fixed with deionized water for 50 mL, and 10 mL of the fixed solution was sucked up and filtered with a 0.45 μm diameter filter membrane with a diameter of 0.45 μm, and the heavy metal content of tea was determined by an inductively coupled plasma mass spectrometer ICP-MS (Thermo Fisher Scientific, USA) (Jing et al. 2019).

### WoE method for ecological risk assessment and determination of relevant indicators

Refer to Wang et al. (2022b) for the WoE method for ecological risk assessment and related formulae.

#### Experiments with tea plants in pots

The test tea seedlings were homogeneous one-year old ‘Longjing 43’ cuttings with a plant height of 15–20 cm, in good health and free from diseases and pests and other undesirable symptoms. Three well-grown, essentially uniform seedlings were implanted in each air-dried soil sample, with three replicates for each treatment, to maintain the same culture conditions. The soil samples were ground, weighed at 1.5 kg, and passed through a 2 mm nylon sieve before being placed into plastic pots. The soil’s water content was then adjusted with deionized water to 70% of the maximum field water holding capacity. Samples were collected to ascertain the pertinent indexes of tea seedlings after 30 days after transplanting.

#### Determination of photosynthetic index of tea plants

Before sampling, a clear and sunny morning between 9:00 and 11:00 was chosen to measure the photosynthetic indexes of the second and third mature leaves of the tea plant using the Li-6400 portable photosynthesizer (LI-COR, USA). LED red and blue light sources were selected to provide effective radiation for photosynthesis of tea plants, and buffer bottles were used to ensure stable air intake, and the buffer bottles were placed in a high place to ensure relatively stable air flow. The parameters were set as follows: Leaf chamber temperature: 25 °C, Flow: 500, Lamp: 1600 μmol m^−2^ s^−1^, Light intensity: 1200 μmol m^−2^ s^−1^. Net photosynthetic rate (*A*), stomatal conductance (*g*_*s*_), intercellular carbon dioxide concentration (*Ci*), and transpiration rate (*E*) were measured.

#### Determination of antioxidant enzyme activities in tea plants

For the determination of antioxidant enzyme activities in leaves of tea seedlings, 0.1 g of fresh sample was ground with 2 mL 0.05 mM phosphate buffer (pH 7.8) in the ice bath. After grinding, the homogenate was centrifuged at 4 °C for 30 min at 10000 rpm, and the supernatant was kept at 4 °C to be tested for enzyme activity. Superoxide dismutase (SOD) activity was determined by measuring the inhibition of the photochemical reduction of pyrogallol utilizing the method of pyrogallol autoxidation method by spectrophotometry at 325 nm (Xu et al. 2019). Catalase (CAT) activity was determined by decomposition of H_2_O_2_ and was measured spectrophotometrically by assessing the decrease in absorbance at 240 nm (Aebi 1984). Peroxidase (POD) activity was determined by the degree of oxidation of guaiacol by spectrophotometer at 470 nm (Xu et al. 2019).

#### Determination of soil microbial function indicators in tea plantations

The urease activity was determined by the sodium phenol-sodium hypochlorite colorimetric method. 5.0 g of air-dried soil samples were weighed and sieved through 0.25 mm sieve, and the samples were incubated with 10 mL of 10% urea as the substrate for 24 h at 37 °C in a constant temperature oven and then filtered. Pipette 1 mL of filtrate was added with 4 mL of sodium phenol and 3 mL of sodium hypochlorite solution, and then shaken well to develop the color, and then volume to 50 mL, and colorimetrically measured at 578 nm within 1 h. The colorimetric analysis was carried out at 578 nm on an enzyme meter (Gu et al. 2009).

Polyphenol oxidase activity was determined by the gallotannin colorimetric method by weighing 1.0 g of air-dried soil samples that had passed through a 0.25 mm sieve, placing them in 50 mL triangular flasks, and then incubating them for 2 h by adding 10 mL of 1% o-catechol solution, so as to allow the o-catechol to be oxidized into purple gallotannin under the action of polyphenol oxidase, and then comparing the colorimetry at 430 nm on an enzyme marker (Ma et al. 2019).

Soil basal respiration was determined with reference to Xu et al. (2019). Briefly, fresh soil samples equivalent to 50 g dry weight in 100 mL beakers were placed in 2.5 L wide-mouth flasks along with beakers containing 25 mL of 1 mol L^−1^ NaOH, and appropriate amounts of distilled water were added every 3 days during the incubation period to maintain the soil samples at a humidity level of 70% of the maximum water-holding capacity. The soil samples were incubated for 42 days at 25 ± 1 °C in darkness. On the 2, 4, 7, 14, 21, 28, 35 and 42 days of incubation, the CO_2_ content was determined by titration method and the cumulative CO_2_ emission was calculated.

### Data analysis

The experimental data were statistically processed using Excel 2019, plotted using ArcMap (v10.3) and R (v4.3.0), and the differences in indicators between different sites were compared using SPSS software applying one-way analysis of variance (ANOVA).

## Result

### Analysis of soil quality in tea plantations

#### Analysis of physical and chemical properties of tea plantation soils

The soil water content of the 34 sites in the study area ranged from 17.10 to 39.78%, with a mean value of 27.77%; pH ranged from 4.22 to 7.30, with a mean value of 5.45; SOM content ranged from 7.82 to 45.17 g kg^−1^, with a mean value of 16.92 g kg^−1^; soil TC content ranged from 4.53 to 36.47 g kg^−1^, with a mean value of 9.96 g kg^−1^. NH_4_^+^-N and NO_3_^−^-N content ranged from 0.20 to 10.32 mg kg^−1^, 1.27 to 47.18 mg kg^−1^, with mean values of 2.29 mg kg^−1^ and 9.13 mg kg^−1^, respectively, while TN content ranged from 0.50 to 2.36 g kg^−1^, with a mean value of 1.01 g kg^−1^. AP and TP content ranged from 0.51 to 235.46 mg kg^−1^ and 202.13 to 2631.15 mg kg^−1^, respectively, with mean values of 79.71 mg kg^−1^ and 794.78 mg kg^−1^. AK and TK content ranged from 61.92 to 292.50 mg kg^−1^, 167.45 to 1296.84 mg kg^−1^, and the mean values were 132.38 mg kg^−1^ and 526.45 mg kg^−1^, respectively. Among them, the NO_3_^−^-N content had the highest coefficient of variation of 106.69%, while soil water content had the lowest coefficient of variation of 0.11% (Table 1).

**Table 1:** Soil properties of 34 samples

Index	Soil moisture (%)	pH	SOM (g kg^−1^)	TC (g kg^−1^)	NH_4_^+^-N (mg kg^−1^)	NO_3_^−^-N (mg kg^−1^)	TN (g kg^−1^)	AP (mg kg^−1^)	TP (g kg^−1^)	AK (mg kg^−1^)	TK (g kg^−1^)
Mean value	27.77	5.45	16.92	9.96	2.29	9.13	1.01	79.71	0.79	132.38	0.53
Minimum value	17.1	4.22	7.82	4.53	0.2	1.27	0.5	0.51	0.2	61.92	0.17
Maximum value	39.78	7.3	45.17	26.2	10.32	47.18	1.92	235.46	2.63	292.5	1.3
Standard deviation	2.24	0.94	7.52	4.34	2.45	9.05	32.96	70.23	0.51	58.61	0.27
Coefficient of variation	0.11%	17.27%	44.47%	43.57%	106.69%	99.15%	32.72%	88.11%	64.61%	44.27%	50.96%

*SOM* soil organic matter, *TC* total carbon, *NH*_*4*_^*+*^*-N* ammonium nitrogen, *NO*_*3*_^*—*^*N* nitrate nitrogen, *TN* total nitrogen, *AP* available phosphorus, *TP* total phosphorus, *AK* available potassium, *TK* total potassium

Based on the China’s agricultural standards “Green food-Environmental quality for production area NY/T 391-2021” (MOA 2021) and “Environmental requirement for growing area of tea NY/T 853-2004” (MOA 2004), as well as soil nutrient diagnostic indexes of ‘3H’ tea plantations, the soil nutrient grading standards of tea plantations were integrated and the sample sites in the study area were classified into soil nutrient grades (Table S1). The percentage of pH up to ‘3H’ tea plantation standard is 47.06%. The proportion of SOM content in grade I – III is 26.47%, 26.47% and 47.06% respectively, the average level was grade II, and 26.47% of them meet ‘3H’. 38.24%, 35.29%, and 26.48% of the sites met the requirements for TN content grade I – III, respectively, with grade I as the mean value level and ‘3H’ compliance at 8.82%. With a mean level of grade I and 79.41% meeting ‘3H’, the percentage of AP content that met the standards for grades I – III were 79.41%, 14.71%, and 5.88%, respectively. The percentage of AK content grade I – III was 67.65%, 32.35%, and 0.00%, with a mean level of grade I and 67.65% conforming to ‘3H’, respectively. It is worth noting that only one site (site 2) had all indicators satisfy the criterion for ‘3H’ tea gardens, while 8 sample sites of tea gardens met the standard of grade II or higher, accounting for 23.53% of all sample sites.

#### Analysis of heavy metal content and spatial distribution of tea plantation soils

**Fig. 2:**
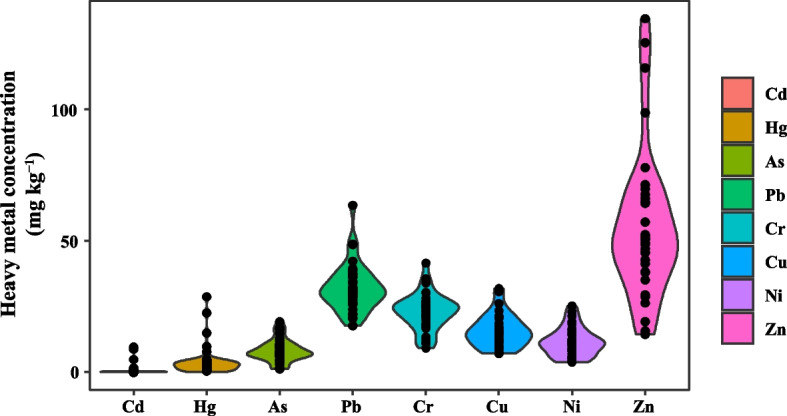
Contents of heavy metals in soil

Soil Cd content ranged from 0.04 to 9.45 mg kg^−1^ with a mean value of 0.84 mg kg^−1^, Hg content ranged from 0.04 to 28.61 mg kg^−1^ with a mean value of 4.54 mg kg^−1^. As Hg ranged from 0.04 to 28.61 mg kg^−1^ with a mean value of 4.54 mg kg^−1^. As ranged from 1.04 to 19.07 mg kg^−1^ with a mean value of 8.73 mg kg^−1^. Pb ranged from 17.56 to 63.49 mg kg^−1^ with a mean value of 32.61 mg kg^−1^. Cr ranged from 9.09 to 41.0 mg kg^−1^ with a mean value of 8.73 mg kg^−1^. The range of Pb content was 17.56 ~ 63.49 mg kg^−1^, and the average value was 32.61 mg kg^−1^. Cr content was 9.09 ~ 41.43 mg kg^−1^, and the average value was 24.69 mg kg^−1^. Cu content ranged from 7.00 to 31.63 mg kg^−1^, and the average value was 16.13 mg kg^−1^. Ni content ranged from 3.70 to 25.08 mg kg^−1^ with a mean value of 12.15 mg kg^−1^. Zn content ranged from 14.18 to 134.62 mg kg^−1^ with a mean value of 58.68 mg kg^−1^ (Fig. 2).

Based on the background values of HM elements in soils of Jiangsu Province as the reference standard values, the percentage of samples with eight HMs in soils of 34 sites exceeding the background values was Hg (91.18%) = Pb (91.18%) > Cd (50.00%) > As (41.18%) > Zn (26.47%) > Ni (0.00%) = Cr (0.00%). Among them, the highest concentrations of As, Cu and Zn were higher than the background values in each site, but their average concentrations were lower than the background values. On the other hand, the average concentrations of Cd, Hg and Pb in the sites were much higher than the background values, reaching 7.64, 28.29 and 1.56 times of the background values, respectively (Table S2).

An essential scale known as coefficient of variation (CV) is used to describe how variable a sample is. CV values below 0.10 indicate mild variability, 0.10 to 0.30 indicate moderate variability, and CV values over 0.30 indicate significant variability. The CVs of these eight HMs in the soils of the test tea plantations were Cd > Hg > As > Zn > Ni > Cu > Cr > Pb in descending order, with the CVs of Cd, Hg, As, Zn, Ni, Cu, and Cr amounting to 266.39%, 139.50%, 52.56%, 51.19%, 47.79%, 39.94%, and 31.03%, respectively (Table S2).

According to the results of the geographical distribution of HMs, the concentrations of Cd are highest in the Dongshan area and lowest in the Xishan area and Northern urban area. The high values of Hg are concentrated in the Xishan area and the southern part of the Dongshan area, and decrease to the north. While high values of As were concentrated in the Dongshan area and the northern part of the Xishan area, and decreased to the south. The highest values of Pb occurred in the Dongshan area, and the concentration was moderate and uniform in the rest of the area. Ni concentration was higher in Xishan area as a whole, and the highest value is in the south of Xishan area. The distribution of Cu concentrations was similar to that of Cd, with high values concentrated in the Dongshan area and lower concentrations in the Xishan area, and high values of Zn concentrated in the northern part of the Xishan area (Fig. S1).

#### Correlation analysis between physical and chemical properties of tea plantation soil and heavy metal content

**Fig. 3:**
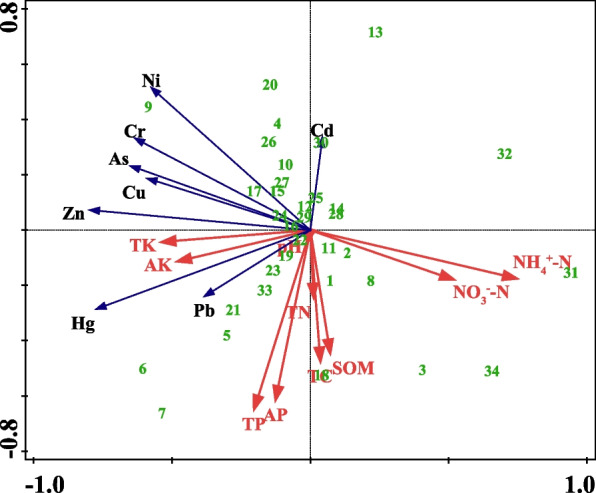
Redundancy analysis plots of soil properties and the heavy metals in soil. SOM, soil organic matter, TC, total carbon, NH4 + -N, ammonium nitrogen, NO3--N, nitrate nitrogen, TN, total nitrogen, AP, available phosphorus, TP, total phosphorus, AK, available potassium, TK, total potassium

The HM contents in tea plantation soils were closely related to their physicochemical properties (Fig. 3). NH_4_^+^-N and NO_3_^−^-N were significantly negatively correlated with seven HMs except Cd, while AK and TK were significantly positively correlated with these seven HMs. Notably, Cd concentration was significantly negatively correlated with SOM, TC, TN, AP, and TP. In the RDA analysis, the individual sample sites were relatively concentrated, and only individual sample sites with higher HM concentrations were far away. In addition, this RDA model could explain 89.37% of the whole environmental variables.

### Analysis of heavy metal content in tea plant

**Fig. 4:**
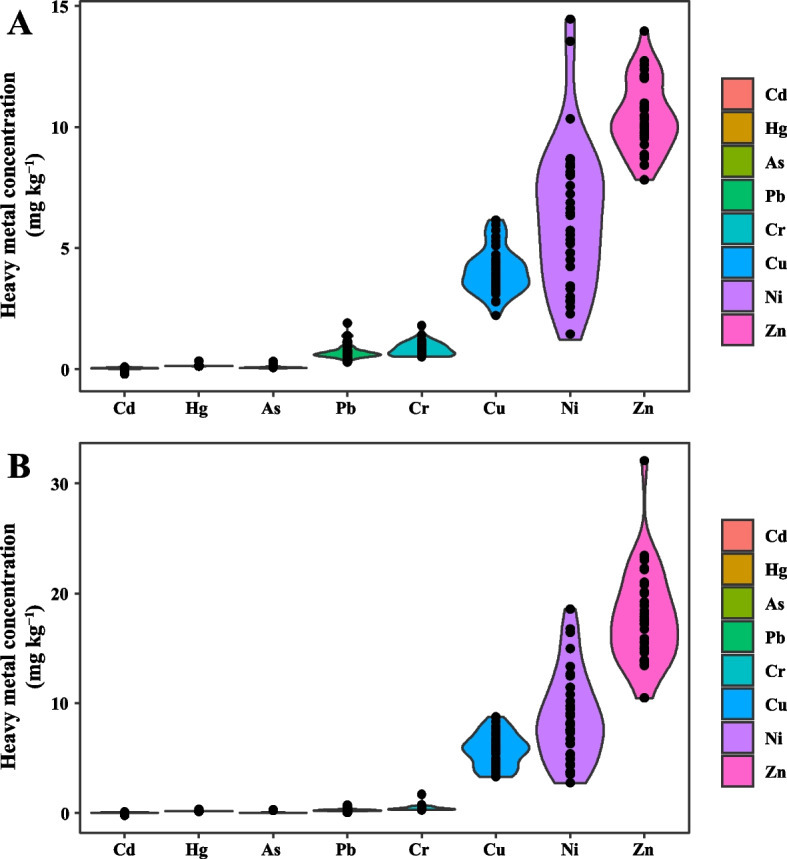
Contents of heavy metals in one bud with three leaves (**A**) and mature leaves (**B**) of tea plant

Considering the standards of ‘Organic Tea NY 5196-2002’ (MOA 2002) and ‘Residue limits for Cr, Cd, Hg, As and F in tea NY 659-2003’ (MOA 2003), 1.0, 0.3, 2.0, 2.0, 5.0 and 30.0 mg kg^−1^ for Cd, Hg, As, Pb, Cr and Cu were selected as the pollution-free tea in the samples. The results showed that the mean values of the six HM elements, Cd, Hg, As, Pb, Cr and Cu, in one bud with three leaves and mature leaves were much less than the limit values, and the overall heavy metal content of tea in this region was at a safe level (Fig. 4, Table S3 and Table S4). The relevant standards of Ni and Zn in tea were not available for the time being, so they were not evaluated.

The CVs of 8 HMs in one bud with three leaves of tea plants were Cd > Ni > Pb > As > Cr > Cu > Zn > Hg in descending order, with the CVs of Cd, Ni, Pb, As, and Cr amounting to 55.09%, 47.62%, 43.14%, 40.86%, and 35.30%, respectively, indicating that there was a high spatial variability of these HMs (Table S3). The high values of Cd, As, Cu, Pb and Zn were scattered in the study area, the high values of Cr were concentrated in the western and southern parts of the Xishan area, Ni concentrations were high in the Xishan area as a whole, and the spatial distribution of Hg was uniform (Fig. S2).

The CVs of the 8 HMs in the mature leaves of tea plants were Cd > Cr > Ni > Pb > As > Cu > Zn > Hg in descending order, among which the coefficients of variation for Cd, Ni, Pb, As, and Cr were 90.03%, 57.05%, 46.61%, 42.06%, and 40.48%, respectively (Table S4), which was similar to that for the distribution of the heavy metal contents in one bud with three leaves. The high value points of Cd were concentrated in the southwest of Xishan area and decreased in the northeast direction, while the Dongshan area gradually decreased from north to south. The high value points of As were concentrated in Xishan area and the overall concentration in Dongshan area was moderate, and the distribution of Pb was similar to that of As. The high value points of Cu, Cr, Ni, and Zn were dispersed in the study area, while the spatial distribution of Hg content was uniform (Fig. S3).

### Characterization of soil-tea heavy metal migration

In this study, the order of bioconcentration factor (BCF) of HM in one bud with three leaves was Ni > Cd > Cu > Hg > Zn > Cr > Pb > As (Table 2), with higher variation in BCF values of Cd, Hg and Ni. The order of BCF values of HM in mature leaves was Ni > Cu > Zn > Hg > Cd > Cr > Pb > As. The content and enrichment coefficients of HM in various organs of tea plants at different sites varied considerably, and the overall performance was as follows: for the four elements of Cd, As, Pb and Cr, one bud with three leaves > mature leaves; for the three elements of Cu, Ni and Zn, mature leaves > one bud with three leaves.

**Table 2:** Bioconcentration factor in soil-tea infusion system

Heavy metal	one bud with three leaves			mature leaves		
	Maximum value	Minimum value	Mean value	Maximum value	Minimum value	Mean value
Cd	186.51%	0.26%	41.42%	81.00%	0.16%	22.77%
Hg	327.24%	0.44%	27.54%	343.24%	0.45%	28.52%
As	11.16%	0.26%	1.42%	1.53%	0.06%	0.43%
Pb	5.31%	0.91%	2.44%	1.88%	0.37%	0.88%
Cr	9.14%	1.31%	4.02%	6.28%	0.99%	2.02%
Cu	71.23%	10.42%	30.67%	85.49%	12.17%	41.89%
Ni	195.54%	21.79%	64.34%	276.50%	20.58%	91.12%
Zn	85.28%	7.39%	24.53%	95.82%	11.05%	39.74%

**Fig. 5:**
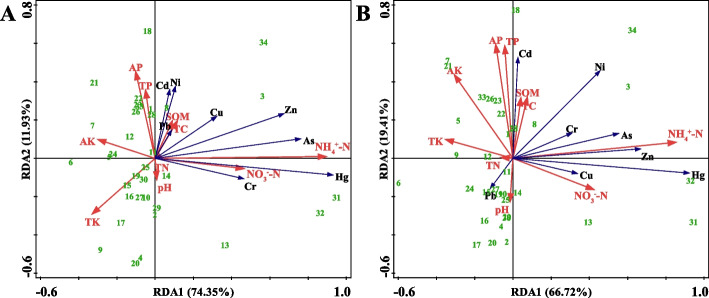
Redundancy analysis plots of soil properties and the bioconcentration factor in one bud with three leaves (**A**) and mature leaves (**B**) of tea plant. SOM, soil organic matter, TC, total carbon, NH_4_^+^-N, ammonium nitrogen, NO_3_^−^-N, nitrate nitrogen, TN, total nitrogen, AP, available phosphorus, TP, total phosphorus, AK, available potassium, TK, total potassium

The HM enrichment coefficients of tea plantation leaves were closely related to the signs of the physicochemical properties of the corresponding Tea plantation soils (Fig. 5). In one bud with three leaves, Hg, As, Cr, Cu and Zn were positively correlated with NH_4_^+^-N and NO_3_^−^-N, while negatively correlated with AK and TK, and Cd, Pb and Ni were positively correlated with SOM, TC, AP and TP, while negatively correlated with pH. This RDA model explains 86.28% of the whole environmental variables. Hg, As, Cr, Cu, and Zn in mature leaves were positively correlated with NH_4_^+^-N and NO_3_^−^-N, while negatively correlated with AK and TK, and Cd was positively correlated with SOM, TC, AP, and TP, while negatively correlated with pH. It is noteworthy that the effect of soil physicochemical properties on the enrichment coefficient of Pb in mature leaves was opposite to that of Cd. This RDA model could explain 86.13% of the whole environmental variables.

### WoE for ecological risk assessment

#### Chemical risk evaluation

**Fig. 6:**
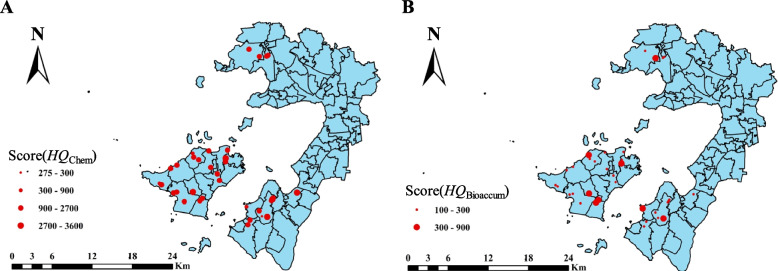
Chemical risk assessment (**A**) and bioaccumulation risk assessment (**B**) for heavy metal of soil in different sites

Based on the relative accumulation index of each total HM, the chemical risk score of the sample sites was calculated (Fig. 6A), and the chemical risk score ranged from 275 to 3525. According to the chemical risk evaluation criteria, only four out of 34 sample sites had chemical risks score below 100 (Table S5), of which 2 sites were between 100 and 300, and 2 sites were between 300 and 900. The chemical risk scores of the other 30 sites were over 900. Therefore, the results of the chemical risk evaluation indicated that the assessment site requires the next level of bioaccumulation risk evaluation.

#### Bioaccumulation risk evaluation

In-situ soil cultivation incubation test was conducted on the soil of the sample site in the study area. After 30 days of incubation, the results of ANOVA analysis of the cumulative concentrations of 8 HMs in the tea plant showed that the differences in the cumulative concentrations of the heavy metal elements, Cd, As, Pb, Cr, Cu, Ni, and Zn, except for Hg, reached a highly significant level (*p* < 0.01) in the tissues of the tea plant at different sites (Table S6).

The soil bioaccumulation risk index of 34 sites in the study area ranged from 200 to 525 (Fig. 6B). According to the bioaccumulation risk evaluation criteria, the bioaccumulation risk scores of 8 sample sites were between 100 and 300, and the rest of the sample sites were ranged from 300 to 900 (Table S5). In terms of risk level, the bioaccumulation risk level of each sample site was generally lower than the chemical risk. In addition, the next level of ecotoxicological risk evaluation was required due to the presence of moderate and higher bioaccumulation risks in the soils at the site.

#### Ecotoxicological risk evaluation

The toxicity test of in situ soil was used in this assessment, and the physiological and biochemical indicators of tea plant were selected as the endpoints for ecotoxicological risk evaluation. The results of ANOVA analysis on the physiological indicators of tea plant showed that the differences of CAT, SOD and POD activity, *A*, *g*_*s*_, *Ci* and *E* between soils at different sites reached highly significant levels (Table S7).

**Fig. 7:**
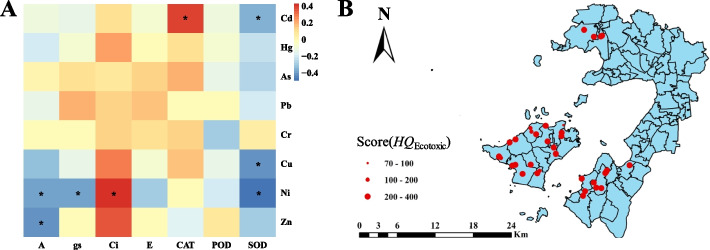
Correlation analysis between physiological indexes of tea plant and heavy metals in soil (**A**) and ecotoxicology risk assessment for heavy metal of soil in different sites (**B**). * means significant correlation at 0.05 level

The correlation analysis between soil HM concentrations and physiological and biochemical indexes of tea plant showed that the *A*, *g*_*s*_ and SOD activity of tea plant were significantly negatively correlated with the concentrations of four HMs, and that the *Ci* and CAT activity were significantly positively correlated with the concentrations of Ni and Cd, respectively (Fig. 7A). The above results indicated that the physiological and biochemical indexes of tea plant were greatly affected by soil HM content, and the toxic effects on tea plant responded to the overall toxicity of soil heavy metals. Therefore, *A*, *g*s, *Ci*, CAT and SOD activity of tea plant were selected as the endpoints of ecotoxicological risk evaluation.

Based on the physiological and biochemical indicators of tea plants, the ecotoxicological risk score of soils at different sample sites was calculated, and the results showed that the range of ecotoxicological risk score was 76–394 (Fig. 7B), and according to the criteria for ecotoxicological risk evaluation, the ecotoxicological risk scores of 5 sample sites were below 200 (Table S8).

#### Ecosystem risk evaluation

ANOVA analysis of soil microbial functional indicators showed that the differences in β-glucosidase (β-Glu) activity, polyphenol oxidase (PPO) activity, urease activity, and soil basal respiration among different sites were all at highly significant levels (Table S7). Soil microbial community functional indicators were highly significantly correlated with soil organic carbon, and each functional indicator was divided with the organic carbon content of the corresponding soil site before exposure-effect analysis to eliminate the masking of HM toxic effects by organic matter.

**Fig. 8:**
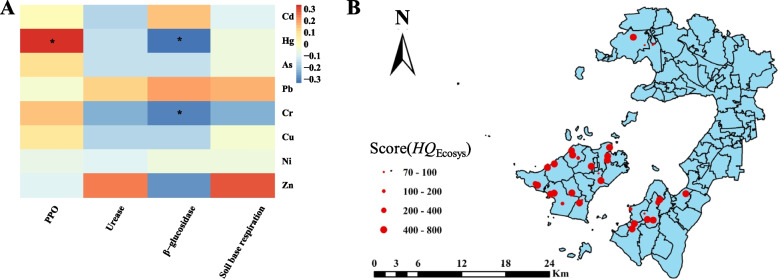
Correlation analysis between microbial functional indicators and heavy metals in soil (**A**) and ecosystem risk assessment for heavy metal of soil in different sites (**B**). * means significant correlation at 0.05 level

The results of Pearson correlation analysis of microbial function indicators with the content and total amount of each HM in the soil showed that PPO activity was significantly positively correlated with Hg content, and β-Glu activity was significantly negatively correlated with Hg and Cr (Fig. 8A). HM Hg is an important influence factor of soil microbial function in the study area, so β-Glu and PPO activity can be used as the endpoint of soil ecosystem risk evaluation in the study area. The results of ecosystem risk evaluation showed that the range of ecosystem risk integral was 70–450 (Fig. 8B). According to the ecosystem risk evaluation criteria, the ecosystem risk scores of 21 sample sites were over 400, and those for 6 sites belonged were below 100 (Table S8).

#### Comprehensive ecological risk characterization

**Fig. 9:**
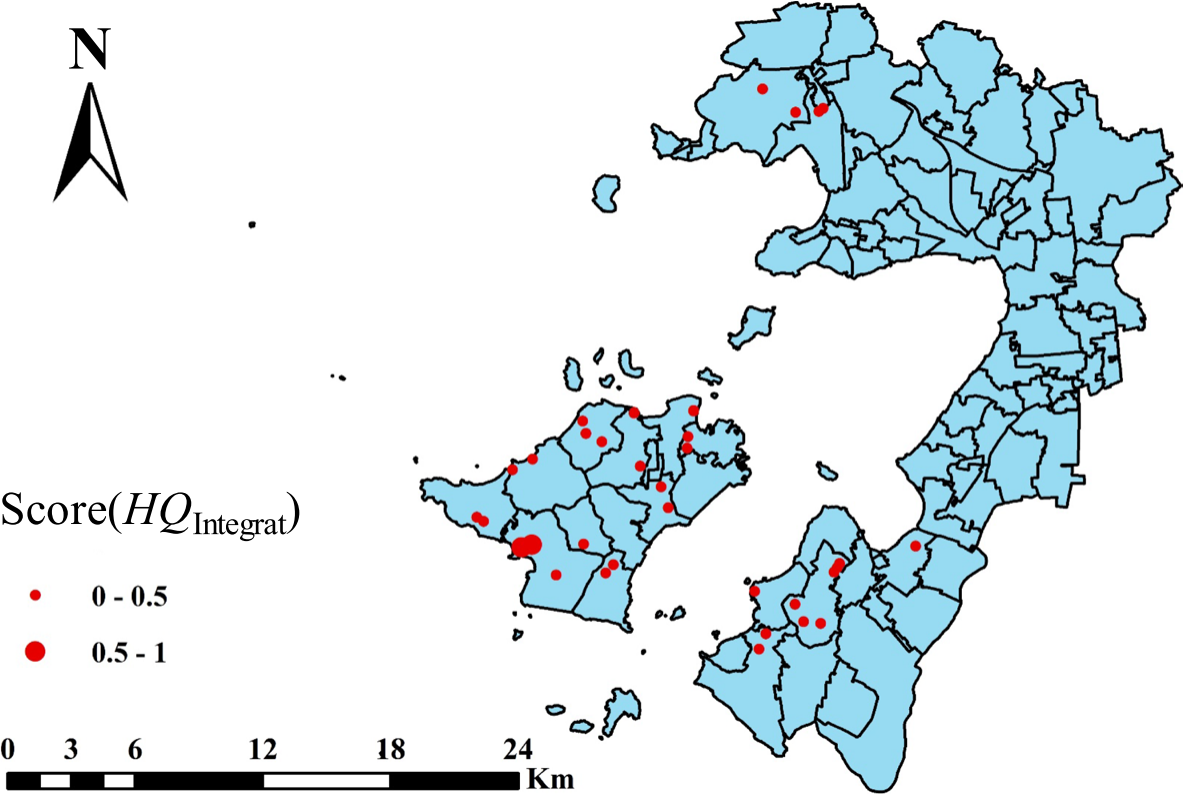
Integrated ecological risk assessment for heavy metal contamination of soil in different sites

The integrated ecological risk index was calculated based on the composite ecological risk index of chemical risk index, bioaccumulation risk index, ecotoxicological risk index, and ecosystem risk index, which ranged from 0.04 to 0.60 (Fig. 9). The standard deviation of the chain of evidence for the comprehensive ecological risk assessment is less than 0.4, so the accuracy of the results of this assessment is high. According to the comprehensive ecological risk assessment criteria (Table S9), the comprehensive ecological risk index of the 32 sites is less than 0.5, which is acceptable as agricultural land.

## Discussion

### Analysis of tea plantation soil quality and heavy metal in tea plants

The average soil Cd content of the tea plantation sites in the study area exceeded the background value in Suzhou, and the spatial variation scale of Cd content in the tea plantations differed greatly, with its coefficient of variation reaching 266.39%, while the spatial correlation was relatively weak, indicating that the content of Cd element in the soil in the study domain was relatively significant by the external interference, and it is necessary to pay close attention to the further development of Cd element in the soil, and to take preventive and control measures at the earliest possible time. In addition, the CVs of Hg, As, Zn, Ni, Cu, and Cr were all more than 30% indicating those HMs among different tea gardens in the study area were more significantly disturbed by the outside world, with greater spatial variability, which might be due to the influence of industrial, transportation and agricultural activities.

The main sources of HM contamination are agricultural activities such as pesticide application, sewage irrigation, misuse of feed additives, etc. (Kersten et al. 2017). On the other hand, transportation pollution is also an important factor contributing to the accumulation of heavy HMs in the soil, and barite accumulates through atmospheric deposition processes (Xu et al. 2021). According to the ecological environment of the tea plantation in the study area, it is presumed that the Cd in the soil of this tea area comes from different degrees of exogenous HM, such as pollution brought about by pesticide and chemical fertilizer application and due to the highway, national highway, provincial highway, and township road running through the pollution caused by automobile exhaust emission. In addition, the mean value of Hg concentration in the 34 sites far exceeded the background value, with a coefficient of variation of 139.50%. Hg may mainly come from the soil parent material, and the study area is located in the Cu, Mo, Au, Ag, Pb, and Zn metallogenic subzone of Xuanzhou-Suzhou, and the associated products of the cinnabar minerals are the main carriers of Hg in the soils, and the two spatial distributions are highly coupled, and the weathering and erosion of rocks in the area are responsible for the high accumulation of Hg in tea plantations in around Tai Lake area in Suzhou (Kuang and Yang 2022). The weathering and erosion of rocks in the region and the leaching and transportation process are the reasons for the high accumulation of Hg in tea plantation soils around Tai Lake in Suzhou (Ziolek et al. 2017). Secondly, dry and wet atmospheric deposition may also be one of the sources of Hg (Liu et al. 2020). Hg elements from fossil fuel combustion, smelting and exhaust emissions from transportation vehicles in human activities accumulate in the soil through atmospheric deposition and air adsorption, thus causing pollution (Liu et al. 2020).

There is a correlation between soil physicochemical properties and HM elements, of which nine physicochemical properties except pH are closely related to soil HM elements, which is basically consistent with the findings of Yang et al. (2018). It is worth noting that pH was not the main factor affecting the heavy metal content in this study, probably because the total amount of HM was determined in this study and pH mainly affects heavy metal effectiveness. When pH decreases, heavy metal ions are more likely to exist in the form of soil solution, and the effectiveness increases; as pH increases, negatively charged soil colloids increase and adsorb metal cations, thus decreasing the effectiveness of HMs (Qu et al. 2020).

Soil Cd and Hg accumulation was high in the test tea plantations, but the Cd and Hg contents in one bud with three leaves and mature leaves of tea plant complied with the relevant standards of pollution-free tea, and the high content of heavy metals in the soil may not necessarily cause the exceeding of the corresponding HM content in the tea leaves, which is in agreement with the results of the study by He and Peng (2023). The possible reason for this phenomenon is that the content of heavy HM in tea is affected by multiple factors, and the content of HM, its morphology, and the external environment may affect the process of transferring heavy metals from the soil to the tea leaves (Jin et al. 2008; Jin et al. 2005; Xu et al. 2018). The bioconcentration coefficient can reflect the size of the plant’s ability to absorb and accumulate HM, and the larger the coefficient, the stronger the plant’s ability to absorb and accumulate HMs. Therefore, the above results indicate that the accumulation capacity of Ni in tea plant leaves is the strongest among the 8 HMs, which may be related to the higher content of Ni in the effective state in the soil (Kanwar et al. 2023). Cd showed a high enrichment capacity overall, which was inconsistent with the results of most studies. Cd was poorly mobile in the tea plant, and the absorbing roots acted as an obvious barrier to prevent the transfer of soil Cd to the new shoots, with most of it being immobilized by the absorbing roots, and a low percentage of it being transported above ground (Shi et al. 2006; Zhang et al. 2020c). The reasons for the differences may be related to the growing environment of tea plants and tea plant varieties. The uptake of Cd by tea plants can not only be obtained directly from the soil through the root system, but also through the uptake channels of other essential micronutrients and the stomata on the surface of leaves into the leaves of tea plants, so that the value of BCF is higher. The overall enrichment coefficient of Hg is low, but it varies a lot. The Hg content of soil ranged from 0.04 to 28.61 mg kg^−1^, while the Hg content of tea plant leaves ranged from 0.11 to 0.13 mg kg^−1^. This indicates that there is no obvious connection between the uptake of Hg from soil by tea plants and the Hg content of soil, and therefore, the Hg enrichment coefficients were lower with a wide range of variation. In addition, the HM contents and enrichment coefficients of various organs of tea plants at different sites varied greatly, and the enrichment capacities of one bud with three leaves and mature leaves for different metal elements had large differences, and their enrichment mechanisms need to be further explored.

### Ecological risk assessment of soil heavy metal in tea plantations

In this study, the WoE method was used to assess the ecological risk of tea plantations soil, including chemical risk, bioaccumulation risk, ecotoxicological risk, and ecosystem risk assessment (Wang et al. 2022b). The results of the chemical risk assessment showed that the chemical risk of most of the sites reached the severe level and above, and the overall risk of the study area was high due to the high accumulation of soil Cd and Hg in the tea plantations for testing. For the Dongshan area, where the high Cd concentration is concentrated, the chemical risk can be reduced by popularizing the use of orchard grass mulching cultivation technology, and intercropping or planting Cd-rich plants such as *Y. erythrocarpa* (Lin et al. 2015), *S. alfredii* (Yang et al. 2004) and *B. niveaon* (Chen et al. 2021) in cultivation. In the western and southern parts of the eastern mountains, where high values of Hg concentration are concentrated, it is recommended that intercropping or set-planting of enrichment plants *P. nummularia*, *B. officinalis*, *A. ageratoides* (Qian et al. 2018), *E. polymnioides* (Chamba et al. 2017), etc. At the same time, with the application of amendments to reduce the effective state content of Cd in the soil. Heavy metal absorption by tea plants is related to their forms, and reducing the effective state content of soil heavy metals is the key to reduce the absorption of heavy metals by tea plants and control the enrichment of heavy metals (Li et al. 2022; Wang et al. 2022a). Relevant studies have proved that the application of amendments such as quicklime with organic fertilizers, calcium superphosphate and organic fertilizers can reduce the content of Cd in the active state in tea plantation soils, and the rate of reduction is higher with time (Lin et al. 2019; Yi et al. 2022).

The overall bioaccumulation risk is good, with only 8 sites reaching the medium level. The reason for the high risk of bioaccumulation in this site is that the tea plant has a strong ability to enrich Cu. Farmers should pay attention to the Cu status of the soil in the tea plantation, and reduce the input of Cu by applying organic fertilizers in a reasonable way, carrying out mixed irrigation and irrigation in a rotation manner, selecting super-accumulative plants for bio-improvement to increase the output of Cu, and improving the buffering property of the soil for Cu by applying calcium and iron in order to avoid the negative impacts of Cu on the tea plant due to its overabundance.

Photosynthesis is the process by which green plants use light energy to produce organic matter, which is an indispensable part of plant growth and development, and can be used to characterize the degree of damage and health of plants under HM stress (Topchiy 2010). Meanwhile, the activities of some major enzymes in the plant antioxidant system as well as some osmoregulatory substances can also be used to characterize the degree of stress in plants, such as SOD, POD, and CAT, etc. SOD, as the first line of defense in the plant enzyme antioxidant system, scavenges a large number of reactive oxygen radicals induced to accumulate by HM stress, which are then used by POD and CAT to break down H_2_O_2_ into water and oxygen through disproportionation (Xu et al. 2019). In order to explore the ecological risk of soil HM in tea plantations, this paper selected various physiological indicators of tea plants, a typical crop in the study area, as the endpoints for ecotoxicological risk evaluation.

Soil microorganisms are important components of ecosystems and are the core of soil ecosystems, widely involved directly or indirectly in regulating the cycling of energy and matter involved in soil ecosystems (Petersen et al. 2012), soil fertility formation (Hadar and Papadopoulou 2012), and so on. Studies have shown that soil microorganisms are more responsive to stresses from various pollutants than plants (Giller et al. 1998). While soil enzymes are closely related to soil microorganisms, many enzymes in the soil are secreted by microorganisms and participate in the cycling of matter and energy in the soil together with microorganisms, so soil enzyme activities are also subject to HM stress. The selection of β-Glu and PPO activities related to soil carbon cycling as ecosystem risk assessment endpoints in this study is inconsistent with Kandeler et al. (1996). This is because the diversity of the soils themselves can cause differences in the degree of HM stress between experiments, and because properties such as soil pH, organic matter content, clay content, and iron and aluminum oxide content can alter the effects of heavy metals on soil microorganisms and the biochemical processes they are involved in, making it difficult to reasonably evaluate the effectiveness of heavy metal stress on soil microorganisms and microbial processes. Therefore, before finalizing the evaluation endpoints, the correlation analysis between the measurement endpoints and the actual contamination characteristics of the site soil was carried out to ensure that the evaluation endpoints had an exposure-response relationship under the actual conditions, which was better targeted for the site assessment. The final screening of this ecosystem effect evaluation endpoints resulted in 2 indicators, β-Glu and PPO activities, being related to the degree of soil pollution in the study area, which affected the reliability of this chain of evidence to a certain extent. Microorganisms are more active in metabolic activities and they absorb and transform organic matter. In the root of tea plant soil, there are many types and numbers of microorganisms, so it is more reasonable to use microorganisms for soil remediation. Such as cyanobacteria, sulfate-reducing bacteria and some algae, can produce extracellular polymers and heavy metal ions to form complexes, thereby improving the fixation efficiency of soil heavy metals.

Based on the results of soil HM content detection and ecological risk assessment of tea plantations in the study area, differentiated governance and restoration measures should be taken. On the one hand, for the tea plantations with more serious ecological risks of soil HM, the first step is to implement orderly withdrawal, and then give full play to the function of ecosystem self-regulation and purification by applying nature-based solutions, while with the help of pollutant-soil nature joint governance technology and nature engineering, according to the spatial differences in HM enrichment, with plant and animal remediation technology as the main and chemical remediation measures as the supplement On the other hand, for all of the HMs in the study area, the soil is treated in the same way as the soil. On the other hand, all the tea plantations in the study area were treated with the help of ecological remediation and ecological restoration. Firstly, drawing on the principles of pollution ecology, we analyze the sources of soil HM in tea plantations, identify the pollutant propagation paths, and take joint control measures at the source, channel and convergence nodes to carry out the soil heavy metal treatment and improvement projects; in addition, through the use of eco-field cans, ditch and river ecology, purification ponds, wastewater treatment, and ecological landscape shaping of farmland and other measures, we can enhance the connectivity of the ecological network of tea plantations, and fully exert the influence of animals such as earthworms on soil heavy metals. Earthworms and other animals swallow and absorb soil heavy HM, plant roots adsorb soil heavy HM, and algae and other microorganisms degrade soil heavy HM, to improve the soil ecological environment and enhance the overall ecological function of the tea plantation system through physical-chemical-biological joint restoration techniques; furthermore, strengthen the construction of the protective forest network, and through the installation of plant protective forest isolation zones, effectively prevent the suburb or the near-tourist area from Excessive vehicle exhaust emissions on the soil heavy metal accumulation situation aggravated, from the source to eliminate the heavy metal enrichment.

## Conclusion

The average pH level of tea plantation soils around Tai Lake in Suzhou is within the range suitable for tea growth, site 2 fully meets the requirements of ‘3H’ tea plantations. HMs in tea plantation around Tai Lake in Suzhou accumulated in different degrees and had obvious regional distribution characteristics. In addition, the HMs content of tea leaf samples was generally at a safe level, which met the requirements of pollution-free tea. The composite ecological risk index evaluation of ‘WoE’ method showed that the soil in the area around Tai Lake in Suzhou is basically safe, and almost all sample sites were the most suitable agricultural land for tea plantations.

### Supplementary Information

**Additional file 1: Fig. S1.** Spatial distributions of contents of heavy metals in soil. **Fig. S2.** Spatial distributions of contents of heavy metals in one bud with three leaves of tea plant. **Fig. S3.** Spatial distributions of contents of heavy metals in mature leaves of tea plant.

**Additional file 2: Table S1.** Grading evaluation results of soil physical-chemical indexes. **Table S2.** Heavy metal concentrations in 34 samples. **Table S3.** Heavy metal concentrations in one bud with three leaves of tea plant. **Table S4.** Heavy metal concentrations in mature leaves of tea plant. **Table S5.** Risk classification for chemical risk and bioaccumulation risk. **Table S6.** Heavy metal accumulation in leaves of tea seedling. **Table S7.** Physiological indexes in leaves of tea seedling. **Table S8.** Soil microbial functional indicators. **Table S9.** Classification of integrated ecological risk index.

## Data Availability

All data and material included in this study are available upon request by contact with the corresponding author.

## Abbreviations

AK: Available potassium
AP: Available phosphorus
BCF: Bioconcentration factor
CV: Coefficient of variation
EC: Electrical conductivity
GPS: Global positioning system
NH_4_^+^-N: Ammonium-N
NO_3_^−^-N: Nitrate-N
RDA: Redundancy analysis
SWC: Soil moisture content
SOM: Soil organic carbon
TC: Total carbon
TK: Total potassium
TN: Total nitrogen
TP: Total phosphorus
