# Supplementary material for: Ecological risk assessment of heavy metals in tea plantation soil around Tai Lake region in Suzhou, China
Source: Stress Biol. 2024 Feb 16;4(1):15. doi: 10.1007/s44154-024-00149-x (PMC10873261; doi:10.1007/s44154-024-00149-x)
Supplement: Supplementary file 1 — Additional file 1: Fig. S1. Spatial distributions of contents of heavy metals in soil. Fig. S2. Spatial distributions of contents of heavy metals in one bud with three leaves of tea plant. Fig. S3. Spatial distributions of contents of heavy metals in mature leaves of tea plant. [file 44154_2024_149_MOESM1_ESM.docx]

**Fig. S1** Spatial distributions of contents of heavy metals in soil.

**Fig.S2** Spatial distributions of contents of heavy metals in one bud with three leaves of tea plant.

**Fig. S3** Spatial distributions of contents of heavy metals in mature leaves of tea plant.
