# Supplementary material for: Ecological risk assessment of heavy metals in tea plantation soil around Tai Lake region in Suzhou, China
Source: Stress Biol. 2024 Feb 16;4(1):15. doi: 10.1007/s44154-024-00149-x (PMC10873261; doi:10.1007/s44154-024-00149-x)
Supplement: Supplementary file 2 — Additional file 2: Table S1. Grading evaluation results of soil physical-chemical indexes. Table S2. Heavy metal concentrations in 34 samples. Table S3. Heavy metal concentrations in one bud with three leaves of tea plant. Table S4. Heavy metal concentrations in mature leaves of tea plant. Table S5. Risk classification for chemical risk and bioaccumulation risk. Table S6. Heavy metal accumulation in leaves of tea seedling. Table S7. Physiological indexes in leaves of tea seedling. Table S8. Soil microbial functional indicators. Table S9. Classification of integrated ecological risk index. [file 44154_2024_149_MOESM2_ESM.docx]

**Table S1** Grading evaluation results of soil physical-chemical indexes

| Index | Level | Range | Number of site | Proportion（%） |
| --- | --- | --- | --- | --- |
| pH | III | - | 18 | 52.94 |
|  | II | - |  |  |
|  | I | - |  |  |
|  | ‘3H’ | 4.4 – 5.5 | 16 | 47.06 |
| SOM（g·kg^−1^） | III | < 15 | 16 | 47.06 |
|  | II | 15 – 20 | 9 | 26.47 |
|  | I | > 20 | 9 | 26.47 |
|  | ‘3H’ | ≥ 20 | 9 | 26.47 |
| TN（g·kg^−1^） | III | < 0.8 | 9 | 26.47 |
|  | II | 0.8 – 1.0 | 12 | 35.29 |
|  | I | > 1.0 | 13 | 38.24 |
|  | ‘3H’ | ≥ 1.5 | 3 | 8.82 |
| AP（mg·kg^−1^） | III | < 5 | 2 | 5.88 |
|  | II | 5 – 20 | 5 | 14.71 |
|  | I | > 20 | 27 | 79.41 |
|  | ‘3H’ | ≥ 20 | 27 | 79.41 |
| AK（mg·kg^−1^） | III | < 60 | 0 | 0.00 |
|  | II | 60 – 100 | 11 | 32.35 |
|  | I | > 100 | 23 | 67.65 |
|  | ‘3H’ | ≥ 100 | 23 | 67.65 |

SOM, soil organic matter, TN, total nitrogen, AP, available phosphorus, AK, available potassium, ‘3H’, high quality, high efficiency and high yield.

**Table S2** Heavy metal concentrations in 34 samples

| Index | Cd | Hg | As | Pb | Cr | Cu | Ni | Zn |
| --- | --- | --- | --- | --- | --- | --- | --- | --- |
| Mean value (mg kg^−1^) | 0.84 | 4.41 | 8.12 | 32.11 | 22.89 | 15.63 | 11.44 | 55.77 |
| Minimum value (mg kg^−1^) | 0.04 | 0.04 | 1.04 | 17.56 | 9.09 | 7.00 | 3.70 | 14.18 |
| Maximum value (mg kg^−1^) | 9.45 | 28.61 | 19.07 | 63.49 | 41.43 | 31.63 | 25.08 | 134.62 |
| Standard deviation (mg kg^−1^) | 2.24 | 6.16 | 4.27 | 9.17 | 7.10 | 6.24 | 5.45 | 28.55 |
| Coefficient of variation (%) | 266.39 | 139.50 | 52.56 | 28.56 | 31.03 | 39.94 | 47.79 | 51.19 |
| Background values (mg kg^−1^) | 0.09 | 0.08 | 9.40 | 22.00 | 75.60 | 23.40 | 32.80 | 64.80 |

Background values in soil at Tai Lake region (Chen 2018).

**Table S3** Heavy metal concentrations in one bud with three leaves of tea plant

| Index | Cd | Hg | As | Pb | Cr | Cu | Ni | Zn |
| --- | --- | --- | --- | --- | --- | --- | --- | --- |
| Mean value (mg kg^−1^) | 0.04 | 0.12 | 0.06 | 0.74 | 0.84 | 4.07 | 6.36 | 10.42 |
| Minimum value (mg kg^−1^) | 0.01 | 0.11 | 0.03 | 0.31 | 0.50 | 2.21 | 1.22 | 7.81 |
| Maximum value (mg kg^−1^) | 0.11 | 0.13 | 0.16 | 1.90 | 1.80 | 6.15 | 14.45 | 13.96 |
| Standard deviation (mg kg^−1^) | 0.02 | 0.00 | 0.02 | 0.32 | 0.30 | 0.94 | 4.08 | 1.47 |
| Coefficient of variation (%) | 55.09 | 2.65 | 40.86 | 43.14 | 35.30 | 23.14 | 47.62 | 14.08 |
| Pollution-free limit (mg kg^−1^) | 1.00 | 0.30 | 2.00 | 2.00 | 5.00 | 30.00 | - | - |

Pollution-free limit in tea is according to ‘Organic Tea (NY 5196-2002)’ (MOA 2002) and ‘Residue limits for chromium, cadmium, mercury, arsenic and fluoride in tea (NY 659-2003)’ (MOA 2003). There are no relevant standards for Ni and Zn in tea, so no evaluation is made.

**Table S4** Heavy metal concentrations in mature leaves of tea plant

| Index | Cd | Hg | As | Pb | Cr | Cu | Ni | Zn |
| --- | --- | --- | --- | --- | --- | --- | --- | --- |
| Mean value (mg kg^−1^) | 0.03 | 0.13 | 0.02 | 0.26 | 0.44 | 5.73 | 8.75 | 17.68 |
| Minimum value (mg kg^−1^) | 0.00 | 0.11 | 0.01 | 0.16 | 0.25 | 3.29 | 2.73 | 10.46 |
| Maximum value (mg kg^−1^) | 0.11 | 0.13 | 0.06 | 0.71 | 1.68 | 8.75 | 18.55 | 32.08 |
| Standard deviation (mg kg^−1^) | 0.02 | 0.00 | 0.01 | 0.11 | 0.25 | 1.51 | 4.08 | 4.33 |
| Coefficient of variation (%) | 90.03 | 3.86 | 40.48 | 42.06 | 57.05 | 26.40 | 46.61 | 24.49 |
| Pollution-free limit (mg kg^−1^) | 1.00 | 0.30 | 2.00 | 2.00 | 5.00 | 30.00 | - | - |

Pollution-free limit in tea is according to ‘Organic Tea (NY 5196-2002)’ (MOA 2002) and ‘Residue limits for chromium, cadmium, mercury, arsenic and fluoride in tea (NY 659-2003)’ (MOA 2003). There are no relevant standards for Ni and Zn in tea, so no evaluation is made.

**TableS5** Risk classification for chemical risk and bioaccumulation risk

| *RTR*_w-Chem_ | Score (*HQ*_Chem_) | Level |
| --- | --- | --- |
| 0 ≤ *RTR*_w-Chem_ < 1.3 | Score (*HQ*_Chem_) < 100 | I |
| 1.3 ≤ *RTR*_w-Chem_ < 2.6 | 100 ≤ Score (*HQ*_Chem_) < 300 | II |
| 2.6 ≤ *RTR*_w-Chem_ < 6.5 | 300 ≤ Score (*HQ*_Chem_) < 900 | III |
| 6.5 ≤ *RTR*_w-Chem_ < 13 | 900 ≤ Score (*HQ*_Chem_) < 2700 | IV |
| 13 ≤ *RTR*_w-Chem_ | 2700 ≤ Score (*HQ*_Chem_) | V |

**Table S6** Heavy metal accumulation in leaves of tea seedling

| Index | Cd | Hg | As | Pb | Cr | Cu | Ni | Zn |
| --- | --- | --- | --- | --- | --- | --- | --- | --- |
| Mean value (mg kg^−1^) | 0.00 | 0.13 | 0.12 | 0.31 | 0.58 | 2.55 | 1.70 | 6.63 |
| Minimum value (mg kg^−1^) | 0.00 | 0.13 | 0.09 | 0.24 | 0.32 | 1.58 | 1.20 | 4.42 |
| Maximum value (mg kg^−1^) | 0.02 | 0.13 | 0.17 | 0.81 | 1.06 | 15.43 | 2.33 | 13.38 |
| Standard deviation (mg kg^−1^) | 0.00 | 0.00 | 0.02 | 0.10 | 0.15 | 2.30 | 0.26 | 1.67 |
| *F*-value | 10.96 | 0.00 | 20.64 | 29.48 | 3.52 | 57.61 | 37.66 | 7.44 |
| *P*-value | 0.00** | 1.00 | 0.00** | 0.00** | 0.00** | 0.00** | 0.00** | 0.00** |

** means significant difference at 0.01 level (One-way ANOVA).

**Table S7** Physiological indexes in leaves of tea seedling

| Index | Ph  (μmol CO_2_ m^-2^ s^-1^) | gs  (mol H_2_O_2_ m^-2^ s^-1^) | Ci  (μL L^-1^) | Tr  (mmol m^-2^ s^-1^) | CAT  (U g^-1^) | POD  (U g^-1^) | SOD  (U g^-1^) |
| --- | --- | --- | --- | --- | --- | --- | --- |
| Mean value | 6.1 | 0.03 | 155.49 | 0.41 | 91.57 | 68.49 | 12.38 |
| Minimum value | 2.31 | 0.01 | 24.26 | 0.08 | 24.36 | 2.89 | 7.4 |
| Maximum value | 10.81 | 0.07 | 298.32 | 1.13 | 262.29 | 380.22 | 28.19 |
| Standard deviation | 2.3 | 0.02 | 77.64 | 0.25 | 59.61 | 81.21 | 4.19 |
| *F*-value | 5716.67 | 17521.41 | 720.45 | 15605.42 | 6.47 | 46.51 | 9.42 |
| *P*-value | 0.00** | 0.00** | 0.00** | 0.00** | 0.00** | 0.00** | 0.00** |

** means significant difference at 0.01 level (One-way ANOVA).

**Table S7** Soil microbial functional indicators

| Index | β-glucosidase  (μg p-Nit·g^-1^·h^-1^) | PPO  (mg gllic cid·g^-1^·h^-1^) | Urease  (mg NH_4_^+^-N·g^-1^) | Soil base respiration  (μmol g^-1^ dry weight) |
| --- | --- | --- | --- | --- |
| Mean value | 350.3 | 0.46 | 0.44 | 0.34 |
| Minimum value | 135.04 | 0.05 | 0.18 | 0.32 |
| Maximum value | 891.24 | 1.96 | 1.88 | 0.37 |
| Standard deviation | 163.41 | 0.39 | 0.33 | 0.01 |
| *F*-value | 9.55 | 7.87 | 196.49 | 19.92 |
| *P*-value | 0.00** | 0.00** | 0.00** | 0.00** |

p-Nit, paranitrophenol , PPO, polyphenol oxidase, NH_4_^+^-N, ammonia nitrogen.** means significant difference at 0.01 level (One-way ANOVA).

**Table S8** Risk classification for ecotoxicology risk and ecosystem risk

| *RTR*_w-Ecotoxic_/*RTR*_w-Ecosys_ | Score (*HQ*_Ecotoxic_)/Score (*HQ*_Ecosys_) | Level |
| --- | --- | --- |
| *RTR*_w_ < 0.7 | Score (*HQ*) < 70 | I |
| 0.7 ≤ *RTR*_w_ < 1 | 70 ≤ Score (*HQ*) < 100 | II |
| 1 ≤*RTR*_w_ < 2 | 100 ≤ Score (*HQ*) < 200 | III |
| 2 ≤ *RTR*_w_ < 3 | 200 ≤ Score (*HQ*) < 400 | IV |
| 3 ≤ *RTR*_w_ | 400 ≤ Score (*HQ*_Chem_) < 800 | V |

**Table S9** Classification of integrated ecological risk index

| Standard deviation  between lines of evidence (D) | Integrated ecological risk index  (*HQ*_Integrat_) | Assessment results |
| --- | --- | --- |
| D<0.4 | 0.00 < *HQ*_Integrat_ ≤ 0.50 | Suitable |
|  | 0.50 < *HQ*_Integrat_ ≤ 0.75 | Acceptable |
|  | 0.75 < *HQ*_Integrat_ ≤ 1.00 | Unacceptable |
| D>0.4 | 0.00 < *HQ*_Integrat_ ≤ 0.25 | Acceptable |
|  | 0.25 < *HQ*_Integrat_ ≤ 1.00 | Unacceptable |
